# Implementing a Genetic Counselor‐Led Model for Hereditary Myeloid Malignancies: A Real‐World Study

**DOI:** 10.1002/cam4.71240

**Published:** 2025-09-12

**Authors:** Madeline VanDerGraaf, Georgianne Younger, Kyle Dillahunt, Jennifer Smith, Athena Puski, Nicole Blum, Hailey Manwiller, Jaime Nagy, Grerk Sutamtewagul, Kittika Poonsombudlert, Moon Ley Tung

**Affiliations:** ^1^ Holden Comprehensive Cancer Center University of Iowa Healthcare Iowa City Iowa USA; ^2^ Division of Medical Genetics and Genomics, Stead Family Department of Pediatrics University of Iowa Iowa City Iowa USA; ^3^ Franciscan Health Indianapolis Indianapolis Indiana USA; ^4^ Ambry Genetics Aliso Viejo California USA; ^5^ Carver College of Medicine University of Iowa Iowa City Iowa USA; ^6^ Division of Hematology, Oncology, and Blood and Marrow Transplantation, Department of Internal Medicine, Carver College of Medicine University of Iowa Iowa City Iowa USA

## Abstract

**Background:**

Hereditary hematological malignancy syndromes (HHMS) are more common than previously thought, and identification of an HHMS syndrome can inform the choice of treatments, transplant, and testing of other family members. Genetic testing guidelines for hematological malignancy have broadened; however, there remain a multitude of barriers and complexities with germline genetic testing for these patients. Here, we describe a process for the evaluation and testing of patients for HHMS as well as our respective findings.

**Methods:**

Adult patients with a new diagnosis or history of myeloid malignancy and referred for genetic counseling from 2020 to 2023 within a single institution were reviewed. Descriptive statistics were performed, and frequency data was gathered for relevant patients.

**Results:**

A total of forty‐nine patients were evaluated by a genetic counselor based on their myeloid malignancy; forty‐three patients underwent genetic testing. Genetic testing revealed an HHMS for six patients, with two additional patients found to have abnormalities on ancillary testing that could not be genetically characterized. Thirty‐five patients met NCCN age‐based criteria for genetic testing; however, this was not mutually exclusive with those diagnosed with HHMS. Inpatient genetic counseling had a median timeline of 53 days from referral to result (range: 32–56.75 days). Outpatient genetic counseling had a median timeline of 96 days from referral to result (range: 64–144 days).

**Conclusion:**

Our proposed process demonstrates an efficient structure for patients with hematological malignancy while supporting the importance of the genetic counselor within the malignant hematology and stem cell transplant teams.

## 1 Introduction

Hereditary hematological malignancy syndromes (HHMS) are broadly defined by the predisposition to hematological malignancies due to germline pathogenic/likely pathogenic variants (PVs). These are predominantly, but not exclusively, characterized by a predisposition to myeloid malignancies. The identification of a hereditary myeloid malignancy syndrome can provide valuable insight into treatment, allogeneic hematopoietic stem cell transplant (HSCT), and/or additional risks to the proband, as well as hematological malignancy risk and screening interventions for family members. Such conditions can be isolated without preceding cytopenia, associated with a pre‐existing hematological disorder, or occur as a congenital disorder with multi‐system organ involvement [1, 2, 3]. Previously, HHMS were considered rare and occurring only in young patients with myeloid malignancy. Recent studies have identified that HHMS are more common than previously thought and can be identified in those with later onset diseases [1]. Studies suggest that potentially 26% of adult patients with hematological malignancy have an underlying genetic cancer predisposition, with 13% specifically having a germline predisposition to hematological malignancy [2]. The yield of testing is reduced to approximately 6% when limiting study patients to only those with myeloid disease [3]. Identification of an HHMS can lead to a better understanding of the individual or their family members' risk for malignancy or other related disease, including additional malignancies. While the lifetime risk for many genes is not yet well established, some are indicated to be up to 90% for *CEBPA* [4, 5, 6] or 35%–40% for *RUNX1* [7]. Additionally, those found to have a congenital multi‐system disorder, such as Telomere Biology Disorders (TBD), may be at risk for pulmonary fibrosis, liver disease, or other comorbidities requiring ongoing specialized surveillance [8]. For HSCT, genetic testing can play a major role in donor selection for those with HHMS who plan to use related donors. There may also be other areas where genetic status can inform HSCT conditioning, such as for those with TBD [9, 10]. Surveillance may also be modified for those with HHMS for more frequent CBC or surveillance bone marrow biopsies, and changes in blood count trend may more frequently warrant a diagnostic marrow compared to those without HHMS [11, 12].

As Trottier and Godley describe, there are a multitude of barriers and complexities involved in completing germline genetic testing in a clinical setting for patients with a hematological malignancy. These include the misconception that germline predispositions to myeloid malignancies are rare and only occur in those at young ages, the requirement of a skin punch biopsy and cell culturing for reliable germline genetic results, the difficulty ordering comprehensive testing, and the potential for additional cost burden to patients [13]. The need for cultured fibroblasts for germline assessment for those with hematological malignancy is due to the likelihood of PVs or loss of heterozygosity within the hematopoietic clone, which would result in a false positive or false negative germline result if testing is performed on bone marrow or white blood cells (the DNA source for blood, saliva, and buccal specimens) [14, 15, 16]. HSCT consideration for patients with a potential hematological malignancy genetic predisposition adds to the complexity in the diagnosis and management of these patients. Those found to have HHMS will require family members to be tested if they are being considered for donation, as using an asymptomatic carrier may increase the risk for donor‐derived leukemias or other adverse outcomes [17, 18, 19]. This requires complex genetic counseling for the recipient and donor as to the implications of the genetic result for family members who are found to have the predisposition. These barriers may lead to an underdiagnosis of hereditary hematological malignancy in clinical settings.

Since 2016, support for germline genetic testing for those with myeloid malignancies has improved through the inclusion of myeloid neoplasms with germline predisposition as a provisional disease entity within the World Health Organization (WHO) and additional recognition in the 2022 5th edition as a classification subtype [20]. Similarly, the International Consensus Classification of Myeloid Neoplasms and Acute Leukemias [21] and the European LeukemiaNet [22] both adopted a classification system within the same year that includes hematologic neoplasms with germline predisposition the same year. Best practice management guidelines for patients with HHMS and other germline malignancy predisposition conditions who are considering HSCT are also available [19]. Additionally, the development of a practice resource for the National Society of Genetic Counselors provides support to genetic counselors (GC) in the risk assessment and evaluation for hematological malignancies [23].

The support for germline genetic testing is also seen in the development of guidelines from the National Comprehensive Cancer Network (NCCN), which now recommends referrals for germline genetic testing for HHMS based on key features. The germline genetic testing criteria are based on the age at diagnosis, distinct pathologies (such as relatively “hypocellular” marrow), syndromic features, personal or family cancer history, or the identification of possible germline variants at suggestive variant allelic frequency (VAF) on genetic tumor profiling. These guidelines aid clinicians through the complex navigation of selecting patients with hematological malignancy for germline genetic testing.

There remains a high degree of diagnostic heterogeneity amongst commercial assays available to assess for HHMS. This complicates the clinical test selection process and may also lead to underdiagnosis of HHMS due to false‐negative test results [24]. The variability in test selection, in combination with the difficulty of testing patients with a hematological malignancy, requires providers to carefully consider the clinical picture, family history, and their structure for testing logistics.

A systematic and efficient process is crucial to coordinate, evaluate, and interpret germline genetic testing for patients with myeloid malignancy within a concise timeline. Previous studies suggest that up to 66% of patients will require a referral for germline genetic testing, with over half being considered urgent [25]. With these challenges in mind, we developed a process for genetic testing for adults with myeloid malignancies to ensure equitable access to comprehensive germline genetic testing for HHMS. Here, we describe our single‐institution process for assessment, evaluation, and coordination of HHMS for adult patients with myeloid malignancy and the relevant findings.

## 2 Methods

### 2.1 Patient Recruitment

**FIGURE 1:**
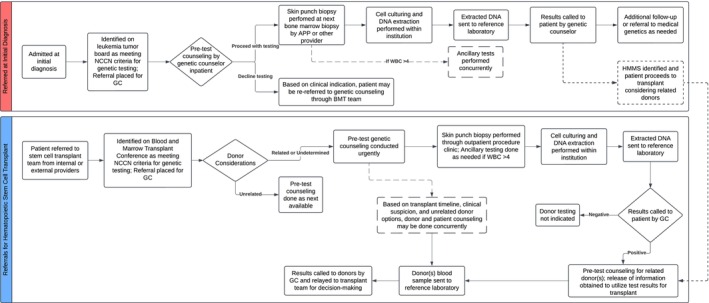
Process for HHMS genetic counseling and testing. APP, advanced practice provider; BMT, blood and marrow transplant; DNA, deoxyribonucleic acid; GC, genetic counselor; HHMS, hereditary hematological malignancy syndrome; NCCN, National Comprehensive Cancer Network; WBC, white blood cell.

A multidisciplinary team of hemato‐oncologists, clinical geneticists, pathologists, and GCs at the University of Iowa Health Care developed a systematic process for HHMS testing of adults with myeloid neoplasms (Figure 1). Criteria for consideration of germline genetic testing were based on NCCN Myelodysplastic Syndrome and Acute Myeloid Leukemia guidelines [26]. Criteria evolved concurrently with NCCN guidelines. Patients were identified through weekly leukemia tumor boards and the HSCT conference lists. Our institution's structure for HSCT conference is for our providers to review every patient referred for or in the process of HSCT. This allows the GC to perform a chart review and determine eligibility for genetic counseling and testing for a high volume of HSCT patients, as well as to identify any donor plan changes for which genetics may become relevant. Providers were informed of the patient's eligibility, and referrals to genetic counseling were placed. This study was approved as a retrospective analysis through the University of Iowa IRB (IRB#: 202210222) with recruitment dates between January 1, 2020, and December 31, 2023.

### 2.2 Proposed Process

Patients identified through this process met with a GC prior to testing. The pre‐test model varied by patient setting, including (a) inpatient counseling while admitted for chemotherapy, (b) counseling coordinated with another outpatient visit, or (c) an independent outpatient visit for genetic counseling only. At the time of the pre‐test counseling, if the genetic testing was deemed urgent due to an upcoming HSCT from a related donor, potential related donors were counseled with parallel genetic testing performed. Genetic test results were disclosed with appropriate post‐test counseling by the GC, with any treatment or HSCT implications discussed with the hemato‐oncologist or the HSCT team. Patients and donors being considered for related HSCT were informed of their results independently of one another, and release of information forms were collected to discuss potential implications of their results on the HSCT process. A subsequent referral to medical genetics for additional clinical evaluation, counseling, and cascade testing was placed if test results indicated an underlying germline predisposition syndrome (e.g., telomere biology disorder). Referrals to medical genetics were also indicated when genetic testing did not identify an underlying predisposition syndrome, but the clinical history or ancillary testing was suspicious. If needed, prior authorizations were submitted by the reference laboratory at the time of sample retrieval, with a few exceptions where the GC submitted on the ordering provider's behalf. A flowchart for the proposed process is provided in Figure 1.

### 2.3 Genetic Studies

A 3–4 mm skin punch biopsy was obtained, with timing dependent on their counseling setting (Figure 1). Most skin fibroblasts were cultured in‐house at the Shivanand R. Patil Cytogenetics and Molecular Laboratory at the University of Iowa. Skin punch biopsies were finely minced, and primary fibroblast cultures were initiated in a T25 flask using AmnioMax (Life Technologies) media. Cells were subcultured to obtain two T25 flasks, which were trypsinized and cells pelleted for DNA extraction, and two additional T25 flasks are cryopreserved for backup. Genetic DNA from cultured fibroblasts was extracted using the DNeasy Blood and Tissue extraction kit (Qiagen #69504). During the extraction procedure, samples were treated with RNase A Solution (Qiagen #158924) to remove RNA. DNA was quantified using a Qubit 4 fluorometer, and an aliquot of DNA was sent to commercial laboratories for HHMS testing. A select few samples were cultured at external commercial laboratories. The selection of the commercial laboratory and the appropriate multi‐gene panel was done at the discretion of the GC in consultation with a clinical geneticist based on a comprehensive personal and family history assessment. Test selection varied over time as laboratory offerings, policies, and NCCN guidelines evolved. All patients with a hematological malignancy who elected to proceed with testing underwent broad multi‐gene NGS panels. Those who were being considered as transplant donors were offered single‐site testing for the familial variant if known or panel testing if being offered testing concurrently or based on additional family history. Variants of uncertain significance (VUS) were evaluated closely, utilizing databases such as ClinVar [27], literature reviews, and other expert organizations in HHMS to gather consensus on variants in HHMS‐related genes. Diagnostic yield was determined by whether a PV was identified in a relevant cancer predisposition gene. This included manifesting heterozygotes with clinical suspicion for the related condition. This does not include unaffected carriers of autosomal recessive conditions. Cascade testing was offered to family members who were interested in genetic testing for the identified variant. Additional surveillance was offered to patients who were identified to have a syndromic condition or solid tumor cancer surveillance, if indicated.

### 2.4 Ancillary Tests

Ancillary tests for inherited bone marrow failure syndromes, such as telomere length measurement and chromosomal breakage analysis, were offered to each patient at the initial consultation. These tests were performed based on clinical suspicion and if the patient's white blood cell counts were at an appropriate level per the reference laboratory standards. Telomere length measurements were performed on peripheral blood using the flow‐FISH methodology at RepeatDx or Johns Hopkins Genetic Laboratory. Chromosomal breakage analysis, either from the peripheral blood or cultured skin fibroblasts, was performed at Cincinnati Children's Hospital. Patients with negative or uncertain genetic sequencing results but suggestive ancillary tests were included in the diagnostic yield for HHMS.

### 2.5 Statistics

Patients' characteristics and timelines were summarized with descriptive statistics to provide an overview of the demographics and clinical data collected.

## 3 Results

### 3.1 Patient Demographics and Characteristics

Between January 1, 2020, and December 31, 2023, a total of 49 patients were seen for genetic counseling based on their myeloid malignancy diagnosis. All were offered germline genetic testing for HHMS on DNA extracted from cultured skin fibroblasts, and 43 completed germline genetic testing with a targeted next generation sequencing panel; six patients declined testing (Table 1). Additionally, two healthy, related donor candidates were tested concurrently with their affected family members. Males and females were equally represented in this study. Approximately three‐quarters of patients had a diagnosis of acute myeloid leukemia (AML), but patients with myelodysplastic syndrome (MDS), chronic myelomonocytic leukemia (CMML) and acute promyelocytic leukemia (APL) were also seen and offered testing. Almost 80% of the patients seen were referred for genetic testing due to their diagnosis of myeloid disease under the age of 50. The median age of diagnosis for the patients tested was 42.7 years old (range: 8–68 years); one patient was diagnosed in childhood but was seen for testing at age 33. Based on NCCN criteria for genetic testing for HHMS, 78% of patients who underwent testing met criteria (Table 1). Genomic studies played an important role in the referral for some patients, particularly those with identified somatic variants in *RUNX1, DDX41*, and *CEBPA*.

**TABLE 1:** Demographics.

	*N* = individuals (%)
Male	25 (51%)
Female	24 (49%)
Mean age at diagnosis	42.7
Outcomes	
Alive	38 (78%)
Deceased	11 (22%)
Transplant status	
After genetic testing	21 (43%)
Prior to genetic testing	4 (8%)
Pending	2 (4%)
Not planned	22 (45%)
Donor source	
Related	5 (19%)
Unrelated	22 (81%)
Counseling setting	
Inpatient	21 (43%)
Outpatient	28 (57%)
Myeloid diagnosis	
AML	37 (76%)
MDS	8 (16%)
APL	3 (6%)
CMML	1 (2%)
Met NCCN age‐based genetic testing criteria (for MDS/AML patients, *n* = 45)	35 (78%)
Testing completed	43 (88%)
Testing declined	6 (12%)
Year patients seen	
2020	4
2021	9
2022	14
2023	22

### 3.2 Germline Genetic Results

Seven patients (16%) were found to have PVs in a hereditary cancer predisposition gene, six (14%) of which were related to HHMS (Table 2). HHMS diagnoses made include Fanconi Anemia due to homozygous intronic PVs in *FANCA* (Patient 2, NM_000135.4: c.283+1G>T), TBD secondary to a heterozygous deletion of *TERC* and exon 1 of the *MECOM* gene (Patient 6, transcript 4), GATA2 deficiency syndrome due to a heterozygous intronic PV in *GATA2* (Patient 19, NM_032638.5: c.1017+572C>T), and a heterozygous PV in *RUNX1* (Patient 26, NM_001754.5: c.553C>T; p.Gln185*). Patient 20 was found to have a heterozygous pathogenic *DDX41* variant shortly before her planned all‐HSCT (NM_016222.4: c.415_418dup; p.Asp140Glyfs*2). This patient's healthy sibling, who was the planned stem cell donor, was tested concurrently and found to carry the same *DDX41* PV. Patient 33 was suspected to have a TBD due to a deletion of the *DKC1* gene, but telomere length studies could not be completed to confirm the diagnosis before she passed away from an aggressive myeloid neoplasm. This patient was also found to be a carrier for Barth syndrome due to a deletion of the *TAZ* gene, and she was suspected to have an X chromosome microdeletion, but this could not be confirmed before her death either. Patient 16 was diagnosed with Hereditary Breast and Ovarian Cancer syndrome due to a heterozygous PV in *BRCA1* (NM_007294.4: c. 1687C>T, p.Gln563*) and had abnormal but indeterminate chromosome breakage analysis results; no overt genetic explanation was identified for the abnormal chromosome breakage analysis. All patients found to have an HHMS were diagnosed with a myeloid neoplasm under age 50, except for the patients with *RUNX1* and *DDX41* PVs, who were diagnosed in their 60s. Five patients were found to be carriers of an autosomal recessive condition (Table 2). A total of 50 VUS were identified in 31 patients. Summaries of these results are shown in Tables S1 and S2.

**TABLE 2:** Genetic testing results.

	*N* = individuals
*Multi‐gene panel testing*	
Overall result interpretation	
Negative	7
VUS	24
Carrier, AR	5
Positive (LPV or PV)	7
Variant classification	
LPV/PV	15
VUS	50
Test failures	
Culturing failure	1 (2%)
NGS failure (del/dup failure)	2 (5%)
Gene	
ADA2	2
BRCA1	1
DDX41	1
FANCA	2^d^
FANCE	1
GATA2	1
MPL	1^c^
RUNX1	1
DKC1	1^a^
TAZ	1^a^
TERC	1^b^
MECOM	1^b^
*Ancillary tests*	
Telomere length analysis (*N* = 6)	
Result	
< 1%ile	2
1%–10%ile	1
Normal	2
Sample failure	1
Chromosome breakage (*N* = 4)	
Result	
Abnormal but indeterminate	1
Normal	3

*Note:* Details on specific variant findings are outlined in Table S2.

Abbreviations: AR, autosomal recessive; LPV, likely pathogenic variant; PV, pathogenic variant; VUS, variant of uncertain significance; XL, x‐linked.

^a^ Large deletion encompassing both genes in the same individual.

^b^ Large deletion encompassing both genes in the same individual.

^c^ Gene associated with condition with autosomal recessive or dominant inheritance; variant specifically identified as related to autosomal recessive disease.

^d^ One individual homozygous, one individual heterozygous.

### 3.3 Ancillary Tests

Four patients successfully completed chromosomal breakage analysis (Table 2). Of these patients, 3 had normal chromosome breakage analysis. One patient's chromosome breakage analysis was abnormal but indeterminate for Fanconi Anemia (Patient 16).

Five patients successfully underwent telomere length analysis (Table 2 and Figure S1). Two of these patients were identified as having telomere length below the first percentile range; one of these patients (Patient 6) was found to have a *TERC* full gene deletion, and the other (Patient 25) remains genetically uncharacterized with only VUS identified on multi‐gene panel testing. One patient was found to have telomere length between the first and tenth percentile range. A sixth patient had telomere length analysis ordered, but testing failed and was unable to be repeated as their HSCT had been completed.

### 3.4 Clinical Outcomes

Thirty‐eight patients are alive as of December 2023, 20 of whom received an HSCT after completion of their testing (Table 1). Of the seven patients found to have HHMS through genetic characterization or abnormal telomere length, four are alive, two of whom have undergone matched unrelated donor HSCT. Three patients with HHMS passed away without HSCT, one due to declining the option of HSCT from an unrelated donor, and two due to progression of disease. Overall, 11 patients are deceased, concluding a mortality rate of 22.4%.

### 3.5 Testing Logistics

#### 3.5.1 Inpatient Timelines

Inpatient counseling and testing timelines showed a median of 7 days from referral to consult, 4 days from consult to skin punch biopsy (coordinated with a day 14 or other bone marrow biopsy), and 36 days from skin punch to return of genetic results. The overall timeline for inpatient counseling from consult to result was a median of 36 days (range: 32–57), and referral to result was a median of 53 days (range: 39–69). Table 3 demonstrates these results with mean, median, and interquartile ranges provided.

**TABLE 3:** Timeline for genetic testing from referral to result across counseling settings.

*N* = days	All			Inpatient			Outpatient		
	Mean	Median	Range	Mean	Median	Range	Mean	Median	Range
Referral to consult^a^	23	14	20	7	7	3	35	25	25
Consult to skin punch	37	3	24	9	4	13	61	0	49
Skin punch to result	52	38	18	58	36	15	47	41	14
Consult to result	89	46	35	66	45	24	108	51	52
Referral to result	111	68	57	73	53	30	145	96	80

*Note:* Declined testing across all patients, *n* = 6. Declined testing from inpatient, *n* = 1. Declined testing from outpatient, *n* = 5.

^a^ Sample size for referral to consult differed to include those who underwent counseling but declined testing.

#### 3.5.2 Outpatient Timelines

Outpatient counseling and testing timelines showed a median of 25 days from referral to consult with no time between consult and skin punch as they were typically coordinated with an outpatient bone marrow biopsy procedure (mean time of 61 days), 41 days from skin punch to result. The overall timeline for outpatient counseling was a median of 51 days from consult to result (range: 38–90) and a median of 96 days from referral to result (range: 64–144). Table 3 demonstrates these results with mean, median, and interquartile ranges provided.

#### 3.5.3 Sample Processing and Volume

Of those tested, only one (2%) had repeated cell culturing failure, which contributed to an increase in result time for that patient. We attempted to utilize a skin punch biopsy for DNA extraction without fibroblast culturing, but the extracted DNA did not meet quality metrics at the reference laboratory. Ultimately, her testing was performed on peripheral blood after achieving remission. Two patients had deletion/duplication analysis fail on panel testing with eventual successful analysis on a second DNA extraction from stored fibroblasts.

As the involved multidisciplinary team became more accustomed to the process, the volume of patients referred for a genetic consultation increased more than 50% each year. Four myeloid patients were seen in 2020, nine in 2021, fourteen in 2022, and twenty‐two in 2023 (Table 2).

## 4 Discussion

Our process facilitates an efficient structure that allows for a genetic testing result within a median timeline of 46 days from the time of the pre‐testing consult, regardless of setting. Average timelines were shorter for inpatient genetic counseling than for outpatient referrals. The facilitation of inpatient genetic counseling and a coordinated skin punch biopsy with their bone marrow biopsy helped to expedite this process and remove barriers for patients. This also allowed the GC to see more patients over time. However, the time needed for culturing and extraction remains the rate‐limiting step.

Our inpatient counseling provided education and consent prior to the patient's next bone marrow biopsy to allow for concurrent skin punch biopsy. The GC was able to prioritize the inpatient consult and perform pre‐test counseling on an average of one week from the time of the referral, with the skin punch biopsy occurring at the next bone marrow biopsy. The flexible time setting in the inpatient unit allowed the GC to conduct the pre‐test counseling and smoothly coordinate the skin punch biopsy in a timely fashion, which has been the key to the expedition of this process in comparison to coordination of care in an outpatient setting. Additional coordination that remains includes procurement of the necessary equipment and supplies, such as culture medium, appropriate interpretation and counseling of the test results, and follow‐up appointments.

The most common reason for timeline outliers includes difficulty coordinating the skin punch biopsy due to a long timeframe before the patient's next visit or patient billing concerns. While culturing and testing failure can also increase time to results, this only occurred for three individuals in our cohort. The utilization of an in‐house culturing facility has allowed for extra samples to be held for re‐testing without an additional biopsy in the event of genetic testing or cell culture failure, which benefitted the two patients who had initial deletion/duplication analysis failure. An additional obstacle encountered in our process was the presence of significant leukopenia, which precluded ancillary testing such as telomere length measurements or chromosome breakage analysis. As telomere length measurement by the flow‐FISH methodology is not validated for skin fibroblasts, patients who have achieved full donor chimerism after HSCT are ineligible for these additional tests if they could not be completed prior. Patient billing concerns were addressed on an individual basis, as each patient's insurance and financial circumstances were vastly different. Some genetic testing laboratories offer financial assistance programs that were discussed with the patients and completed as eligible to help reduce potential costs. For some patients, the GC would initiate a prior authorization as described. However, this was not necessary for every insurance.

Our experiences demonstrate a unique need for genetic counseling and testing for HSCT donors with an HHMS. There remains a theoretical concern for increased risk of donor‐derived leukemia or other adverse outcomes when using asymptomatic related donors who may carry HHMS. For this reason, screening of related donors is recommended when HHMS is identified [19]. In our cohort, two related donors were tested concurrently along with their affected family members for screening purposes prior to HSCT. One patient referred to our institution for HSCT with external work‐up and treatment had a PV in *DDX41* identified on our in‐house molecular profiling panel. The near 50% VAF for this finding raised concerns for germline origin. The initial molecular profiling performed externally did not include analysis of *DDX41*. An urgent genetic counseling referral was placed, and the patient and her sibling were counseled and tested concurrently. Due to her risk for relapse, additional chemotherapy was required while testing was pending. Her sibling's testing revealed the same germline *DDX41* PV, which resulted in the cancellation of the HSCT. This patient then declined all unrelated HSCT donor options that were available and offered to her by the care team. This case suggests careful attention is needed when interpreting somatic profiling and that having a genetic counselor integrated into the HSCT team can prevent further delays in care. The other patient‐sibling pair yielded negative genetic testing results and therefore allowed for the match‐related donor HSCT to proceed as planned. Our institution's standard practice is to run unrelated donor searches concurrently with tissue typing for siblings in the event a back‐up unrelated donor is needed to prevent further delays.

The data from our cohort support previous studies that suggest germline predisposition to myeloid neoplasms may be more common than previously thought. At our single institution, we had a yield of 14% (6/43) for patients with MDS or AML who were found to have a PV predisposing to myeloid disease (Fanconi Anemia, *DKC1*, *DDX41, GATA2, TERC*, and *RUNX1*). One patient had abnormal (less than 1%ile) telomere lengths without genetic characterization. Another patient was found to have a single PV in *BRCA1*, which explained her young‐onset bilateral breast cancer. She also had abnormal but indeterminate chromosome breakage, concerning for Fanconi Anemia or other DNA repair syndrome, with no identified genetic explanation. Our patients with abnormal ancillary studies highlight the importance of these studies when blood counts allow, as well as the need for ongoing research in gene discovery.

Our cohort also presented with challenges in result interpretations, such as the previously described abnormal but indeterminate chromosome breakage, telomere length analysis in the 1st–10th percentile for a patient with a VUS in *TINF2*, and the original classification of a full gene deletion of *DKC1* as a VUS in a female patient who appeared to be a manifesting carrier for Dyskeratosis Congenita, as seen in previous studies due to skewed X‐inactivation [28, 29, 30]. Clinical correlation and case review with a medical geneticist were crucial in determining the best approach for these complex patients. If possible, they were referred for consultation with the medical geneticist; however, many had advanced disease and were unable to attend an additional appointment. Lastly, a recontact challenge was faced when the original uncertain finding of the *DKC1* full gene deletion was upgraded to a pathogenic variant after the patient had expired. Given our experience with this case, we recommend that patients be counseled and advised to nominate a specific next of kin for the return of results in the event they are deceased.

Age was an important factor in the consideration of genetic testing, with most patients seen meeting age‐based NCCN criteria. However, age alone cannot reliably identify those at risk for an HHMS—two patients who received a genetic diagnosis of HHMS in our cohort (*DDX41* and *RUNX1*) were over age fifty at the time of their AML/MDS diagnosis. These patients, as well as other patients who had tested negative, met the criteria through the identification of their germline PV on somatic testing. VUS were common with broad panel genetic testing, especially when including genes with preliminary evidence for disease. Where available, ancillary testing, functional studies, and segregation analyses could benefit the interpretation of uncertain findings related to HHMS. Additional research on the pathogenicity of variants in HHMS is needed to aid in the reclassification of VUS.

Future directions to improve efficiency would include the collection of skin punch biopsy and initiation of culturing at the first contact with the patient. For new onset AML, this would require a skin punch biopsy at the time of the diagnostic bone marrow biopsy. While this would expedite the skin fibroblast culturing process, new challenges may result. First, this would require a genetic counselor to be present at the time of initial diagnosis, which may not be realistic for the standard outpatient genetic counselor practice. If a skin punch biopsy were to be taken prior to counseling, it may result in an unnecessary procedure if the patient elects to cancel the genetic testing, or it is determined from their pathology that they are not indicated for testing. This would also require the hemato‐oncologist to be very familiar with changing guidelines for eligible patients if a genetic counselor is not available. This may also not be the ideal time for discussion of genetics, as the patient may not retain the genetics education with the delivery of a leukemia diagnosis. This also would not be possible for those who are referred for genetics due to pathological or molecular findings. For those being evaluated for HSCT, collecting a skin punch biopsy at initial HLA tissue typing could be considered. This would also require a genetic counselor at the time of initial testing or result in similar unnecessary procedures if the patient declines or is determined not to meet the HHMS criteria. This concept would be feasible if the genetic counselor were well integrated within the HSCT team and counseling coordinated with initial visits, rather than follow‐up or procedure visits. Insurance coverage and patient billing concerns remain a barrier regardless of counseling until HHMS can be more broadly recognized by payers. Thorough pre‐test counseling by a GC for all financial and testing options is strongly encouraged. Our cohort also only represents patients with myeloid malignancy. Other HHMS may be identified by including a broader scope of hematological malignancies.

As discussed, there remain a number of barriers described in our study and others for evaluation for HHMS [13, 25]. In many institutions, access to a GC may be the biggest limitation to implementing such a process. Some possible solutions would include utilizing genetic testing laboratories, genetic counseling services, online genetic counseling services, or offering telemedicine or inpatient genetic counseling services within an institution. As genomic profiling on disease tissue becomes more comprehensive and readily available, there will be more potential germline PVs identified warranting the need for a process for evaluation.

Rapidly expanding guidelines for HHMS suggest the need for integration of genetic counseling and testing within the hematological malignancy diagnosis and care. Whilst this was a retrospective analysis, the standardized process created by the HHMS working group allowed for a smooth referral process to pre‐test and post‐test genetic counseling and germline genetic testing.

## Author Contributions

**Madeline VanDerGraaf:** investigation, data curation, writing – review and editing, writing – original draft, project administration, conceptualization. **Georgianne Younger:** writing – review and editing, data curation. **Kyle Dillahunt:** writing – review and editing, data curation. **Jennifer Smith:** conceptualization, writing – review and editing, data curation. **Athena Puski:** conceptualization, writing – review and editing, data curation. **Nicole Blum:** writing – review and editing, data curation. **Hailey Manwiller:** writing – review and editing, data curation. **Jaime Nagy:** writing – review and editing, methodology. **Grerk Sutamtewagul:** writing – review and editing. **Kittika Poonsombudlert:** writing – review and editing. **Moon Ley Tung:** conceptualization, writing – review and editing, data curation, supervision.

## Conflicts of Interest

The authors declare no conflicts of interest.

## Supporting information


**Table S1:** cam471240‐sup‐0001‐TableS1.docx.
**Figure S1:** cam471240‐sup‐0001‐TableS1.docx.

**Table S2:** cam471240‐sup‐0002‐TableS2.xlsx.

## Data Availability

All data and results generated are available in the article. Additional requests should be directed to the corresponding author via email.
