# Supplementary material for: Implementing a Genetic Counselor‐Led Model for Hereditary Myeloid Malignancies: A Real‐World Study
Source: Cancer Med. 2025 Sep 12;14(18):e71240. doi: 10.1002/cam4.71240 (PMC12426481; doi:10.1002/cam4.71240)
Supplement: Supplementary file 1 — Table S1: cam471240‐sup‐0001‐TableS1.docx. Figure S1: cam471240‐sup‐0001‐TableS1.docx. [file CAM4-14-e71240-s002.docx]

| Supplementary Table 1. Patients identified to have HHMS through genetic characterization or abnormal ancillary testing. | | | | | | | | | | |
| --- | --- | --- | --- | --- | --- | --- | --- | --- | --- | --- |
| Ident-ifier | Sex | Age at Myeloid Diagnosis, Diagnosis | Clinical History | Initial Myeloid Pathology | Family History | Gene with PV Identified (Zygosity) | variant (c./p.) | Other VUS Identified (c./p.) | Ancillary Testing Performed (Result) | Status |
| 2 | F | 33, MDS | Frequent infections, lifelong easy bleeding, short stature (4'2) ((-2.9 SD from Guatemalan female average (4'10'')), hyperpigmentation, nail dystrophy, delayed puberty | **Bone Marrow Exam:** Myelodysplastic syndrome with multilineage dysplasia in a hypercellular bone marrow (95%) with trilineage dyspoiesis, a decreased myeloid to erythroid ratio, and 4% blasts. **Cytogenetics:** 46,XX,der(11)t(1;11)(q21;q23)[4]/46,XX,add(1)(q32),der(11)t(1;11)(q21;q23),add(18)(p11.2),add(19)(q13.3)[16] **FISH:** Copy loss of MLL [11q23] (94.7%) **Somatic Profiling**: *TET2* c.3030G>T 50.3% | **Children and Siblings:** Niece with aplastic anemia at age 5 | FANCA (homozygous) | c.283+1G>T | - | None | Deceased, no transplant |
| 6 | M | 46, MDS | Thrombocytopenia diagnosed at age 25, presumable aplastic anemia followed by MDS diagnosis at age 46, interstitial lung disease diagnosed at age 48 during HSCT workup. Reportedly premature graying at age 16, frequent ear infections, tooth anomaly of "another bone growing from root of tooth" | **Bone Marrow Exam:** Myelodysplastic syndrome, unclassifiable (MDS-U) in a hypocellular bone marrow (30%) with trilineage hematopoiesis including decreased megakaryocytes and 1% blasts. **Cytogenetics:** 46,XY,+1,der(1;14)(q10;q10)[7]/46,XY[13] **FISH:** MECOM [3q26] atypical breakapart rearrangement with 5'*MECOM* loss (84%); RUNX1/RUNX1T1 [21q22/8q22] copy gain (3.3%) **Somatic Profiling:** *ZRSR2* c.904T>C 50% | **Maternal:** Uncle with metastatic cancer of unknown primary, deceased within 3 months of diagnosis | TERC (heterozygous) MECOM (heterozygous) | Full gene deletion of TERC; Deletion of Exon1 of transcript variant 4 (MECOM) | SRP74 (c.18C>G, p.Ser6Arg) | Telomere Length Analysis (<1%ile) | Deceased, no transplant |
| 16 | F | 31, AML | History of AML diagnosed at age 31, underwent unrelated allo-HSCT, stature 5'2". Referred to genetics at age 44 due to multiple primary cancer diagnoses:  **- Bilateral breast cancer** (right: invasive ductal carcinoma, ER/PR-; left: invasive metaplastic matrix producing carcinoma, ER/PR-) with metastasis to the axilla **- Metastatic esophageal squamous cell carcinoma** **- Tonsillar squamous cell carcinoma** | **Bone Marrow Exam:** Acute myeloid leukemia with myelodysplasia-related changes in a hypercellular bone marrow (100%) showing decreased megakaryocytes, marked dyserthropoiesis, dysgranulopoiesis, and 63% blasts. **Cytogenetics:** 46,XX,add(2)(p11.2),del(7)(q22q32),add(11)(p15)[20] **FISH:** None **Somatic Profiling:** None | **Paternal**: Father with esophageal cancer at 65, deceased **Maternal:** Grandmother, grandfather with cancers NOS; first cousin with liver cancer | BRCA1 (heterozygous) | c.1687C>T (p.Gln563*) | MBD4 (c.572C>T, p.Pro191Leu) | Chromosome Breakage Analysis on cultured fibroblasts (Abnormal but indeterminate)  Telomeres not possible due to HSCT | Deceased, transplant prior to testing |
| 19 | F | 38, AML | Melanoma at age 20 with multiple pre-cancerous skin lesions | **Bone Marrow Exam:** Acute myeloid leukemia in a hypercellular bone marrow (95%) with severely decreased trilineage hematopoiesis, and 86% blasts. **Cytogenetics:** 46,XX[20] **FISH:** Normal **Somatic Profiling:** *IDH2* c.419G>A 48.3%; *NPM1* c.860_863dupTCTG 47.3%; *ASXL1* c.4201G>A 48.9% | **Paternal:** First cousin with AML diagnosed at 20 and deceased at 21, grandfather with a cancer NOS, aunt with colon cancer at 52 **Maternal:** Distant relative with breast cancer under 50 | GATA2 (heterozygous) | c.1017+572C>T (Intronic) | ERCC4 (p.Arg576Ser) FANCE (c.1018G>C, p.Gly340Arg) POLE (c.5606A>G, p.Asn1869Ser) | None | Alive, unrelated transplant completed |
| 20 | F | 63, AML | History of IgG lambda MGUS, severe cervical dysplasia after pregnancy | **Bone Marrow Exam:** Acute myeloid leukemia with normocellular marrow (30-40%) with 21.8% blasts on aspirate smear. Residual trilineage hematopoiesis with dyserthropoiesis and increased storage iron with 15% ring sideroblasts. **Cytogenetics:** 46,XX[20] **FISH:** Normal **Somatic Profiling:** *ASXL1* c.1934dup 31.3%; *DNMT3A* c.2645G>A 47.2%; *IDH2* c.515G>A 28.8%; External initial somatic profiling did NOT include analysis of *DDX41* Within institution bone marrow biopsy prior to transplant revealed *DDX41* c.415_418dupGATG 51% | **Children and Siblings:** Brother with 4 melanomas, brother with 1 melanoma, brother with autoimmune disease, daughter with new onset bleeding issues **Maternal:** mother with cervical cancer and melanoma **Paternal:** aunt with breast cancer in her 20's, deceased | DDX41 (heterozygous) | c.415_418dup (p.Asp140Glyfs*2) |  | None | Alive, no transplant |
| 25 | F | 45, MDS | History of multiple abnormal pap smears with two LEEP procedures, HPV+, chronic thrombocytopenia since age 31, lifelong easy bleeding, history of two miscarriages, large area of hypopigmentation and texture changes on back, pectus excavatum, degenerative disc disease diagnosed at age 15, one incidence of acute pancreatitis at age 28, periodontal disease with very little enamel on teeth in childhood, deciduous teeth persisting into adulthood resulting in dentures since 20's **Work-up after genetic testing:** Increased median stiffness of the liver, pulmonary function test showed low normal DLCO (corrected for anemia) | **Bone Marrow Exam:** Hypocellular bone marrow (40%) with preserved granulopoiesis, erythropoiesis showing mild dyserythropoiesis with 7% ring sideroblasts, severely decreased megakaryopoiesis showing dysplastic forms and no increase in blasts.  **Cytogenetics:** 48,XX,+8,+16[5]/46,XX[15] **FISH:** CBFB [16q22] A gain of CBFB from +16 (7.7%); RUNX1T1/RUNX1 [8q22/21q22] A gain of RUNX1T1 from +8 (7.7%) **Somatic Profiling:** *SF3B1* c.1998G>C 3.88% | **Children and Siblings:** Daughter with low platelets at birth and kidney abscesses, brother with an artificial heart valve  **Maternal:** Maternal reportedly lung cancer with "genetic form", aunt with platelet issues, autoimmune disease, and easy bleeding, aunt with cervical cancer with metastasis, grandmother with brain cancer **Paternal:** Uncle with prostate cancer | None | - | MPL (c.1102G>T, p.Val368Leu)  SLX4 (c.996G>T, p.Gln332His) | Chromosome Breakage (normal) Telomere Length Analysis (<1%ile) | Alive, unrelated transplant completed |
| 26 | F | 68, MDS | Chronic thrombocytopenia since age 28 with a lifelong history of easy bleeding and bruising | **Bone Marrow Exam:** Hypercellular bone marrow (40 to 50%) with trilineage hematopoesis, mild dysmegakaryopoiesis and no increase in blasts (1%) **Cytogenetics:** 46, XX **FISH:** Not done **Somatic Profiling:** *NRAS* c.35G>C 5%; *PHF6* c.112_113dup 22.2%; *RUNX1* c.653C>T 50.1% | **Children and Siblings:** Daughter bruises easily, sister with a history of eczema/psoriasis **Paternal:** Aunt with colon cancer >50, grandmother with breast cancer >50 | RUNX1 (heterozygous) | c.553C>T (p.Gln185*) |  | None | Alive, no transplant |
| 33 | F | 41, AML | History of frequent ear infections secondary to a cholesteatoma in teens, underwent a tympanomastoidectomy at 16, reported brittle nails and oral leukoplakia, 5'0" Complex MDS/MPN initial diagnosis with progression to AML | **Bone Marrow Exam:** Hypercellular bone marrow (80-90%) showing trilineage hematopoiesis with markedly increased granulopoiesis, relatively decreased erythropoiesis and megakaryopoiesis, focal slight reticulin fibrosis (MF-1 of 3) and no increase in blasts (<1% by CD34 immunostain). **Cytogenetics:** 46,XX[20] **FISH:** NUP98 [11p15.4] Loss of 3'NUP98 (89%) **Somatic Profiling:** *RUNX1* c.538_539insCTCCT 31.8%; *WT1* c.1102_1109delGTGCGACGinsTTCCCTC 38% | **Siblings and Children:** Sister with kidney and heart disease who passed at age 48 **Maternal:** Mother with heart disease who began greying at age 35, grandmother with autoimmune disease and liver cirrhosis | DKC1 (heterozgous) TAZ (heterozygous) | Full gene deletion of *DKC1* and *TAZ* | FANCI (c.545+3A>T) | None | Deceased, no transplant |


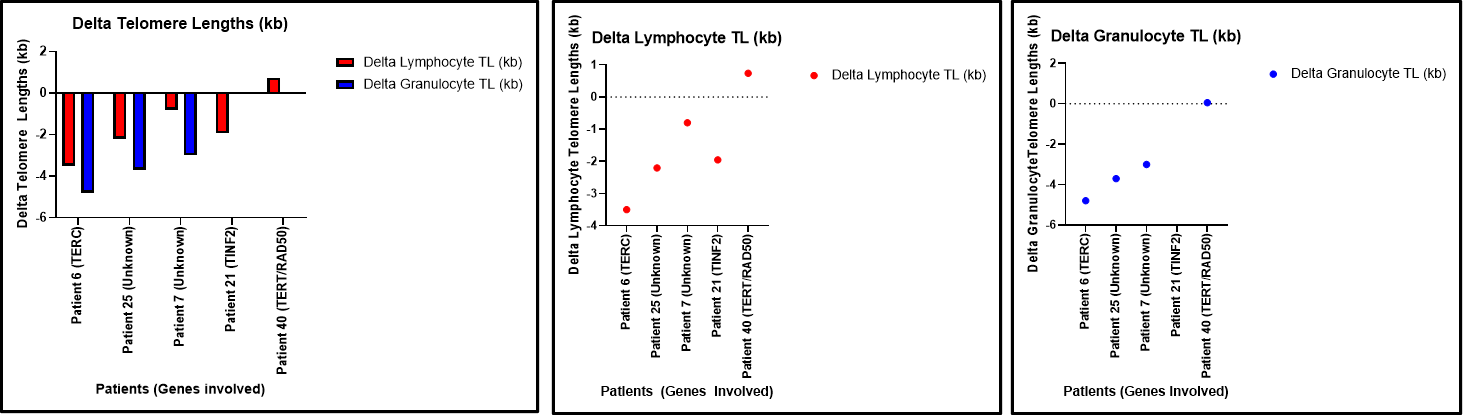
 Supplementary Figure 1. Delta telomere lengths (Flow-FISH methodology) in five patients and their associated gene variants.

Please see attached excel sheet for Supplementary Table 2.
